
JOURNAL INFORMATION
==============================
NLM Title Abbreviation: Innov Aging
Journal ID: Innov Aging
NLM Journal ID: 101703706
Journal ID: innovateage

Innovation in Aging

EISSN: 2399-5300
Publisher: Oxford University Press

ARTICLE INFORMATION
==============================
PMCID: PMC6840647
DOI: 10.1093/geroni/igz038.1052
Article ID: igz038.1052
Article version: 1
Subjects: Session 1355 (Poster), Family Caregiving I

CAREGIVING EXPOSURE AND COGNITION: A PROPENSITY-MATCHED, 10-YEAR FOLLOW-UP

Marino Victoria R 1
Jester Dylan J 2
Haley William E 1
1 University of South Florida , Tampa, Florida, United States
2 School of Aging Studies, University of South Florida , Tampa, Florida, United States
Publication date: 2019 Nov
Electronic publication date: 2019 Nov 8
Volume: 3
Issue: Suppl 1
Issue title: Program Abstracts from the GSA 2019 Annual Scientific Meeting “Strength in Age—Harnessing the Power of Networks”
First page: S285
Last page: S285
Copyright: © The Author(s) 2019. Published by Oxford University Press on behalf of The Gerontological Society of America.
Copyright year: 2019
License: This is an Open Access article distributed under the terms of the Creative Commons Attribution License (http://creativecommons.org/licenses/by/4.0/), which permits unrestricted reuse, distribution, and reproduction in any medium, provided the original work is properly cited.
License URL: https://creativecommons.org/licenses/by/4.0/

Page count: 1

==============================
### CAREGIVING EXPOSURE AND COGNITION: A PROPENSITY-MATCHED, 10-YEAR FOLLOW-UP

Victoria R. Marino,[1] Dylan J. Jester,[2] and William E. Haley[1], *1. University of South Florida, Tampa, Florida, United States, 2. School of Aging Studies, University of South Florida, Tampa, Florida, United States*

Evidence on the effect of caregiving on cognitive health is mixed. Literature suggests that highly-strained subgroups of CGs experience impairment especially related to depression and negative health behaviors. Conversely, others suggest that sustained higher amounts of caregiving is associated with better cognitive functioning over time. This study compared baseline level and 10-year change in executive function (EF) and episodic memory (EM) in middle aged adults with no caregiving exposure, short (less than one year), average (one to less than five years), and long (five or more years) caregiving exposure. Data came from waves 2 and 3 of the Midlife in the United States study. We statistically matched 265 CGs to 265 non-CGs on baseline sex, age, race, education, marital status, and physical health and controlled for depression and anxiety in analyses. Mixed models estimated fixed-effects of time, depression, and a time by caregiving exposure interaction relative to no caregiving exposure. For EF, CGs with short exposure had higher baseline scores relative to non-CGs ($\beta$=.294;p=.023). Though each group declined over time ($\beta$=.298;p<.001), caregiving exposure was not related to the rate of decline in EF. Baseline levels of EM were highest in CGs with average exposure ($\beta$ =.521;p=.011) and long exposure ($\beta$=.482;p =.017). However, not all participants declined in EM over 10 years; CGs with long exposure exhibited the only statistically significant decline ($\beta$ =-.262;p=.026). Results suggest that CG duration is intimately related to EF and EM at baseline, but perhaps only to rate of decline for EM.
